# *SLC30A8* Gene rs13266634 C/T Polymorphism in Children with Type 1 Diabetes in Tamil Nadu, India

**DOI:** 10.4274/jcrpe.galenos.2018.2018.0195

**Published:** 2019-02-20

**Authors:** Ramasamy Thirunavukkarasu, Arthur Joseph Asirvatham, Ayyappan Chitra, Mariakuttikan Jayalakshmi

**Affiliations:** 1 Madurai Kamaraj University, School of Biological Sciences, Department of Immunology, Madurai, India; 2 Government Rajaji Hospital, Clinic of Diabetology, Madurai, India; 3 Government Rajaji Hospital, Institute of Child Health and Research Centre, Madurai, India

**Keywords:** Type 1 diabetes, auto-antigen, polymorphisms, zinc transporter 8 autoantibody, meta-analysis

## Abstract

**Objective:**

Zinc transporter 8 (ZnT8) is a multi-transmembrane protein situated in the insulin secretory granule of the islets of β-cells and is identified as a novel auto-antigen in type 1 diabetes (T1D). The gene coding for ZnT8, solute carrier family 30 member 8 (*SLC30A8*) is located on chromosome 8q24.11. This study aimed to identify the association of *SLC30A8* rs13266634 C/T gene polymorphism with T1D in a sample of T1D children in Tamil Nadu, India.

**Methods:**

The family based study was conducted in 121 T1D patients and 214 of their family members as controls. The *SLC30A8* gene rs13266634 C/T polymorphism was evaluated by polymerase chain reaction-restriction fragment length polymorphism.

**Results:**

No significant differences were observed in either allele (odds ratio: 0.92; confidence interval: 0.33-2.58; p=0.88) and genotype (CC: p=0.74; CT: p=0.82; TT: p=0.80) frequencies of rs13266634 C/T between T1D patients and controls. Transmission disequilibrium test has identified over-transmission of mutant T allele from parents to affected children (T: U=9:7) without statistical significance. Metaanalysis on the overall effects of rs13266634 C allele frequency was not different (p=0.10 and P_heterogeneity_=0.99) in T1D patients as compared to the controls.

**Conclusion:**

The present study along with the meta-analysis does not show any substantial association of the rs13266634 C/T polymorphism with T1D development in this population.

**What is already known on this topic?**

*SLC30A8* rs13266634 C/T polymorphism in type 1 diabetes (T1D) patients from four different populations was previously reported. This gene polymorphism is associated with T1D in the German population, but not in Danish, Japanese and British populations.

**What this study adds?**

To our knowledge, this is the first family-based report addressing *SLC30A8* gene polymorphism in South Indian patients. The present study and the meta-analysis show that the rs13266634 C/T polymorphism is not associated with type 1 diabetes in this population.

## Introduction

Type 1 diabetes (T1D) is a complex, multifactorial disease caused by the selective destruction of insulin-producing pancreatic β-cells (1,2). The autoimmune destruction of pancreatic β-cells by pathogenic T cells predominately targets a number of well-known β-cell auto-antigens (3). Islet cell auto-antigens identified in T1D are Zinc transporter 8 (ZnT8), glutamic acid decarboxylase 65, tyrosine phosphatase-related molecules-2 and insulin (4). ZnT8 is a multi-transmembrane protein, belonging to the family of zinc transporters, having a role in the transport of zinc ions generated from the cytoplasm to the insulin vesicles and plays a major role in insulin maturation (5). During the process of insulin biosynthesis and secretion, frequent exocytosis of glucose stimulated insulin secretion increase the chance of ZnT8 expression on the β-cell surface (6), which further causes more ZnT8 antigen to be exposed. Once ZnT8 is exposed, it can trigger or exacerbate the production of ZnT8 autoantibodies in genetically susceptible individuals (7). Previous studies have reported autoantibodies to ZnT8 to be highly prevalent among new-onset T1D children and have suggested that they could be a marker for disease risk (8,9,10,11). The cation efflux transporter ZnT8 may influence the development of ZnT8 immunogenicity and the phenotypic features of T1D. The solute carrier family 30 member 8 (*SLC30A8*) gene, located in chromosome 8q24.11, encodes for the ZnT8 auto-antigen and comprises 369 amino acids (12,13). Notably, aa268-369 of the cytoplasmic domain of ZnT8, especially ZnT8-325R and ZnT8-325W, is the dominant epitope in T1D. A common non-synonymous single-nucleotide polymorphism (SNP) of *SLC30A8* rs13266634 (C/T polymorphism) encodes either arginine (R) by the C allele or tryptophan (W) by the T allele at aa325 of ZnT8 (14) suggesting that rs13266634 SNP might be critical for humoral autoimmunity in T1D (11,15). Thus, the present study is based on the evidence that *SLC30A8* gene polymorphism is involved in T1D development. The objective of this study was to investigate the association between rs13266634 C/T gene polymorphism and T1D among the children of Tamil Nadu and to apply these results in a meta-analysis to reveal the association between the *SLC30A8* risk allele and T1D for comparison in different ethnic groups.

## Methods

### Subjects

The study subjects comprised 121 T1D patients from the Department of Diabetology, Government Rajaji Hospital in Madurai, Tamil Nadu, India, along with 214 their first degree relatives (120 parents and 94 siblings) as controls. All patients were evaluated by clinical history and routine laboratory tests. The patients met the revised criteria of the American Diabetes Association (ADA) for the screening of T1D (16). Genomic DNA was extracted from 5 mL of peripheral blood sample by salting out method (17). 

Ethic board consent for the study was approved by the Institutional Ethics Committees of Govt. Rajaji Hospital (Ref. No. 23339/E4/3/10) and Madurai Kamaraj University (MKU/IRB/11/11) and consented in writing by the participants.

### Genotype Analysis

Subjects were genotyped for rs13266634 C/T polymorphism of *SLC30A8* gene by polymerase chain reaction (PCR)-restriction fragment length polymorphism (18,19). The region surrounding the polymorphism was amplified with the following primers: Forward, 5’-GGACAGAAAGAGTTCCCATAGCG-3’; Reverse, 5’-ATAGCAGCATGTTTGAAGGTGGC-3’. PCR was performed at 95 °C for 5 minutes, followed by 40 cycles at 94 °C for 40 seconds and 69 °C for 45 seconds. A final extension step was carried out at 72 °C for 5 minutes. The PCR products were digested using enzyme MSp1 (Thermo Scientific, USA) incubated at 37 °C for 4 hours and visualized on 2% agarose gel. In the wild-type genotype (CC) the fragments obtained were of 234 and 195 bp. In the heterozygote genotype (CT), three fragments were detected of 429, 234 and 195 bp. Only one fragment of 429 bp was identified in the homozygote genotype (TT).

### Meta-analysis

An extensive literature search was done to examine the association between T1D and *SLC30A8* gene. The original data were collected from the following electronic databases: PubMed, Elsevier, Science Direct, Web of Science and Google Scholar with key words “Zinc transporter protein member 8, ZnT8, *SLC30A8* gene polymorphism, *SLC30A8* or *SLC30A8* variant, combined with autoimmunity, autoimmune diabetes, T1D mellitus”. All searches were done independently by more than two research investigators. The following inclusion criteria were applied: 1) studies should be case-controlled; and 2) all patients should meet the diagnostic criteria for T1D according to the ADA. Studies were excluded if they did not report on genotype frequency or if they had insufficient data.

### Statistical Analysis

The obtained clinical data were subjected to Student t-test and χ^2^ test after segregating the data based on age, number and sex of the subjects. Odds ratio (OR) and their p-values were calculated by logistic regression, which was performed using STATA 14v software (STATA Corporation, College road, TX, USA). In addition, the transmission/disequilibrium test (TDT) was employed to detect preferential transmission from heterozygous parents to affected offspring (20). The TDT analysis was done by Haploview 4.2v. software (Broad Institute, Cambridge, MA, USA). The level of significance was set at p<0.05. Heterogeneity evaluation was performed by the Cochran’s Q-test (21) and p<0.10 was considered statistically significant. If not significant, OR and 95% confident interval (CI) was estimated by fixed effect model (22), otherwise the random effect model was used (23). Heterogeneity of the data was quantified using the I^2^ test (24). I^2^ value of 25%, 50% and 75% were nominally considered low, moderate and high estimates, respectively. Funnel plot and Egger’s linear regression test was used for the analysis of publication bias (25). Meta-analysis was performed with Rev Man 5.0v. software (RevMan 5.0, The Cochrane Collaboration, Oxford, UK).

## Results

The demographic details of the T1D subjects and controls are given in Table 1. There was no significant differences observed in allele (OR=0.92; CI=0.33-2.58; p=0.88) and genotype (CC: OR=0.92; CI=0.58-1.47; p=0.74; CT: OR=1.05; CI=0.64-1.71; p=0.82; TT: OR=1.13; CI=0.42-3.00; p=0.80) frequencies of rs13266634 C/T between T1D patients and controls, respectively (Table 2). Upon analysis of 30 parent-offspring trios (one affected child and both parents) of the study cohort, TDT analysis identified over-transmission of mutant T allele of rs13266634 C/T polymorphism from parents to affected children (T: U=9:7; MAF=0.194; χ^2^=0.25; p=0.61) without statistical significance.

Meta-analysis of the data via literature survey was able to retrieve 18 studies. Of these, nine were excluded after screening the abstracts, review and irrelevant subject matter. Three studies did not provide comprehensive information. Two studies were not considered as they provided insufficient genotype frequencies. The remaining four studies (14,26,27,28) associated with rs13266634 C/T polymorphism in the *SLC30A8* gene of T1D, which met the required criteria, were included in the present meta-analysis. Along with the present study, a total of five eligible studies with a total of 10,376 T1D patients and 10,027 control subjects were included in the meta-analysis. 

**Figure 1:**
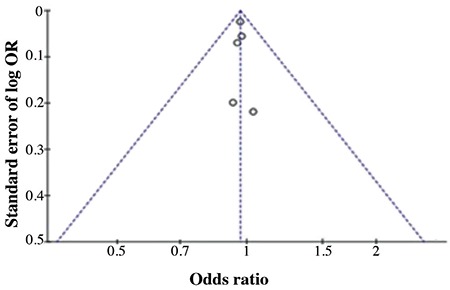
Begg’s funnel plot of *SLC30A8* rs13266634 C/T with type 1 diabetes patients included in this meta-analysis

Characteristics of the said studies and the distribution of rs13266634 C/T genotypes and alleles in T1D patients and controls are given in Table 3. Overall effects of rs13266634 C allele frequency in T1D patients (OR=0.97; CI=0.92-1.01; p=0.10) based on pooled analysis were not different from the controls (Table 4). There was no evidence of virtual asymmetry (χ^2^= 0.29; I^2^=0%; P_heterogeneity_= 0.99) which indicated that no publication bias crept in the meta-analysis (Figure 1). 

In the Forest plot the area of squares, horizontal lines and diamond shows the weight of specific study, confidence intervals and the summary of fixed-effects OR, respectively (Table 4).

In the Funnel plot the open circle represents various studies considered for this plot correlation (Figure 1). No evidence of publication bias was found.

## Discussion

ZnT8 is highly expressed in the pancreatic islet β-cells and recognized as one of the four major auto-antigens in T1D patients. It has been observed that autoantibodies are generated against ZnT8 prior to the onset of disease. It is known that rs13266634 C/T SNP is responsible for the autoimmune response to ZnT8 (12). The rs13266634 C/T plays a susceptibility role in the presence of impaired, autoimmunity-mediated β-cell dysfunction which leads to T1D development (13). Studies of the role of rs13266634 C/T polymorphism in T1D among a global population are scanty. This work appears to be the first family based TDT analysis on rs13266634 SNP with its allele transmission from parents to offspring. As for TDT results, the present study documents over-transmission of mutant T allele of rs13266634 in T1D. In a case control scenario, the present study indicates that there is a lack of association of rs13266634 C/T polymorphism to T1D. A few earlier studies also lent support to this contention in the Danish, Japanese and British populations (14,26,28). However, a German study indicates a higher occurrence of the C allele and CC genotype of rs13266634 C/T polymorphism in early onset of T1D patients compared to controls (27). A recent study revealed that an adjacent locus of rs2466293 in the *SLC30A8* gene seems to predispose to the risk of T1D in individuals of non-European descent (29).

Until now, several publications have investigated the correlation of rs13266634 C/T polymorphisms with T1D (14,26,27,28). However, the results remain inconclusive. In order to reach a more concrete opinion of this contentious matter, a meta-analysis was performed with expanded sample size, aiming to explore the relationship of polymorphism at rs13266634 C/T of the *SLC30A8* gene with susceptibility to T1D. However the result of the meta-analysis indicated that the C allele conferred no risk in the development of T1D. Nevertheless, we should point out that one of the previous meta-analyses on T2D revealed that the rs13266634 C/T polymorphism is significantly associated with impaired glucose tolerance (30).

### Study Limitations

The study is limited by a relatively small number of subjects. Varied studies from different ethnicities with large sample size are required to conclusively confirm the role of rs13266634 C/T polymorphism in T1D.

## Conclusion

This result demonstrates that the allele, genotype, genetic models and allele transmission of rs13266634 C/T polymorphism are not strongly associated with T1D in the children of a Tamil Nadu population. The meta-analysis also indicates that the rs13266634 C/T polymorphism was not associated with T1D.

## Figures and Tables

**Table 1:**
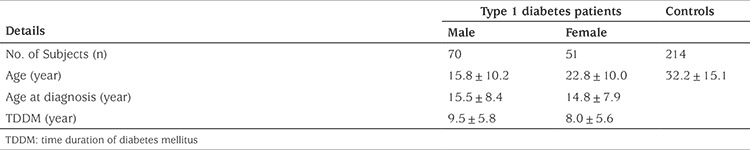
Demographic details of the type 1 diabetes patients and controls

**Table 2:**
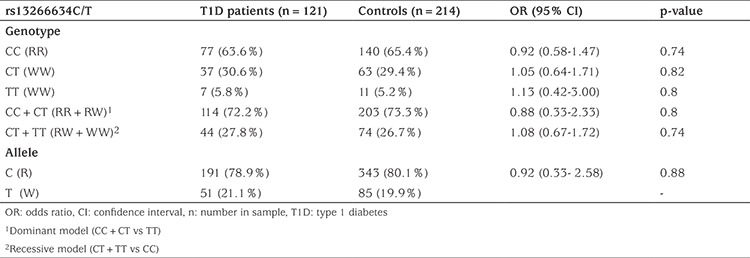
*SLC30A8* rs13266634C/T genotypes and allele frequencies in type 1 diabetes patients and healthy controls

**Table 3:**
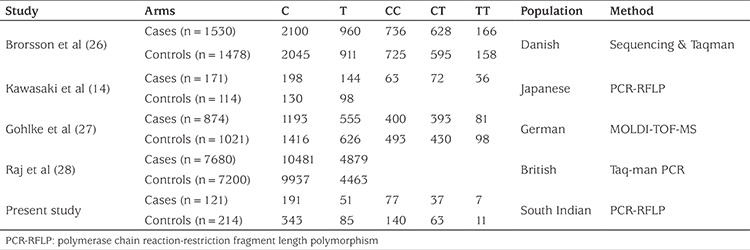
Distribution of *SLC30A8* genotype and allele among type 1 diabetes patients and controls included in the meta-analysis

**Table 4:**
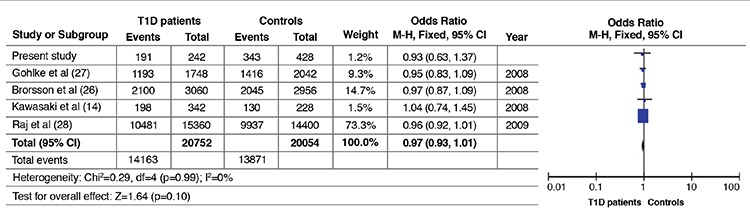
Forest plot depicting the association of *SLC30A8* rs13266634 C-allele in type 1 diabetes
